# Development of Cost-Effective SNP Markers for Genetic Variation Analysis and Variety Identification in Cultivated Pears (*Pyrus* spp.)

**DOI:** 10.3390/plants13182600

**Published:** 2024-09-18

**Authors:** Jae-Hun Heo, Jeyun Yeon, Jin-Kee Jung, Il Sheob Shin, Sung-Chur Sim

**Affiliations:** 1 Department of Bioindustry and Bioresource Engineering, Sejong University, Seoul 05006, Republic of Korea; 2 Seed Testing and Research Center, Korea Seed & Variety Service, Gimcheon 39660, Republic of Korea; 3 Pear Research Center, National Institute of Horticultural & Herbal Science, Rural Development Administration, Naju 58216, Republic of Korea

**Keywords:** fruit crop, molecular marker, genotyping by sequencing, plant variety protection, breeding

## Abstract

Pear (*Pyrus* spp.) is a major fruit crop in the Rosaceae family, and extensive efforts have been undertaken to develop elite varieties. With advances in genome sequencing technologies, single-nucleotide polymorphisms (SNPs) are commonly used as DNA markers in crop species. In this study, a large-scale discovery of SNPs was conducted using genotyping by sequencing in a collection of 48 cultivated pear accessions. A total of 256,538 confident SNPs were found on 17 chromosomes, and 288 SNPs were filtered based on polymorphic information content, heterozygosity rate, and genome distribution. This subset of SNPs was used to genotype an additional 144 accessions, consisting of *P. pyrifolia* (53), *P. ussuriensis* (27), *P. bretschneideri* (19), *P. communis* (26), interspecific hybrids (14), and others (5). The 232 SNPs with reliable polymorphisms revealed genetic variations between and within species in the 192 pear accessions. The Asian species (*P. pyrifolia*, *P. ussuriensis*, and *P. bretschneideri*) and interspecific hybrids were genetically differentiated from the European species (*P. communis*). Furthermore, the *P. pyrifolia* population showed higher genetic diversity relative to the other populations. The 232 SNPs and four subsets (192, 96, 48, and 24 SNPs) were assessed for variety identification. The 192 SNP subset identified 173 (90.1%) of 192 accessions, which was comparable to 175 (91.1%) from the 232 SNPs. The other three subsets showed 81.8% (24 SNPs) to 87.5% (96 SNPs) identification rates. The resulting SNPs will be a useful resource to investigate genetic variations and develop an efficient DNA barcoding system for variety identification in cultivated pears.

## 1. Introduction

Pear (*Pyrus* spp.) is widely cultivated in temperate regions as a major member of the Rosaceae family, which includes economically important fruit crops such as apple (*Malus* spp.), peach (*Prunus persica* (L.) Batsch), and cherry (*Prunus avium* L.). The *Pyrus* genus consists of at least 22 species, including 5 major cultivated species, *P. communis* L., *P. pyrifolia* (Burm.) Nak., *P. ussuriensis* Maxim., *P. bretschneideri* Rehder, and *P. sinkiangensis* Yu, T.T. [1,2]. Of these, *P. communis* is a European pear species, while the other four species are known as Asian pears that are mostly cultivated in China, Korea, and Japan [3]. The cultivated species are diploid (2n = 2x = 34) and have high levels of heterozygosity with widespread cross ability. In addition, this fruit crop has narrow morphological diversity between and within species, and thus the phenotype-based assessment of genetic variation is often difficult in pears.

A large amount of effort has been put forth to develop new varieties every year in pear breeding programs. Since plant breeding requires a costly and time-consuming process, it is important to protect breeders’ intellectual property rights. A plant variety protection (PVP) system was established by the International Union for the Protection of New Varieties of Plants (UPOV) and has been used in 79 member countries and organizations (as of February 2023). In the PVP system, the distinctness, uniformity, and stability (DUS) test, which is required to register a new variety, is conducted based on phenotypic evaluations for a large number of morphological traits during two growing seasons, which is laborious [4]. Thus, use of molecular markers has been considered for the DUS test in the biochemical and molecular techniques (BMT) working group of the UPOV [5,6]. High-throughput genome sequencing and genotyping technologies have facilitated marker development to improve the efficiency of the DUS test [4,7].

Single-nucleotide polymorphisms (SNPs) are the most abundant variants in DNA sequences, and several automated platforms are available for high-throughput genotyping. With these advantages, SNPs have been commonly used as co-dominant markers for genetic analyses. Next-generation sequencing (NGS) has facilitated SNP discovery for population genetics, quantitative trait loci (QTL) mapping, and genome-wide association study (GWAS) in crop species [8]. For pears, 15,146 genome-wide SNPs were generated from genotyping by sequencing (GBS) and used to investigate genetic variations and directional selection in the 214 accessions, consisting of Asian, European, and interspecific hybrid pears [9]. Genetic diversity and population structure were also studied in germplasm collections using large numbers of SNPs across genomes from GBS (10,186 SNPs) and resequencing (over 2.4 million SNPs) [10,11]. Moreover, SNPs have been used to identify QTL associated with traits of interest [12,13,14,15].

Although genome-wide SNPs have been identified and utilized in the previous studies, cost-effective SNPs are still required for DNA barcoding in cultivated pears representing the Asian and European species. Therefore, the present study aimed to develop core SNP markers for variety identification using a collection of 192 cultivated pear accessions. With this objective, 256,538 genome-wide SNPs were generated with a GBS approach in a subset of 48 pear accessions including *P. pyrifolia* and interspecific hybrids. Of these, 288 SNPs were selected to genotype an additional 144 accessions with the Fluidigm platform. Genetic variations between and within the pear species were assessed using the resulting genotypic data. Furthermore, we generated four marker sets (192, 96, 48, and 24 SNPs) and evaluated their performances for variety identification. The genome-wide SNPs and marker sets generated in this study will facilitate the development of a cost-effective and accurate DNA barcoding system for DUS testing and thus benefit breeders by protecting their ownership of new elite varieties in pear breeding programs.

## 2. Results

### 2.1. Genome-Wide SNP Discovery and Fluidigm Genotyping

Genotyping by sequencing (GBS) in the 48 pear accessions generated a total of 696.2 million reads ranging from 2.3 million to 73.7 million per accession with an average of 14.5 million (Table 1 and Table S1). Of these, 647.9 million reads (97.9%) were mapped to the *P. pyrifolia* genome with an average depth of 22.84 reads. The reads represented an average of 57.4 Mb for each accession that is 0.1 × coverage for the reference genome assembly v1.0 (503.8 Mb) of *P. pyrifolia* [16]. With the mapped reads, a total of 2,493,326 SNPs were identified, and 256,538 SNPs were filtered based on minor allele frequency (>5%), missing data rate (<15%), and minimum depth (3×) for further analysis.

The pruned SNPs showed uneven distributions across 17 chromosomes, ranging from 9541 (chromosome 4) to 23,827 (chromosome 15) (Table 2). As a subset, 478 SNPs were selected with three criteria: >0.25 of the polymorphic information content (PIC) value, <35% of the heterozygosity rate, and a physical position relative to the *P. pyrifolia* genome. These SNPs were distributed across 17 chromosomes with the intervals ranging from 0.01 Mb (chromosome 12) to 7.35 Mb (chromosome 5) (Table 2 and Figure S1). Based on the reference genome annotation, 326 SNPs (68.2%) were coding sequence variants, while the other 152 SNPs (31.8%) were found in intron (96 SNPs) and intergenic (56 SNPs) sequences (Table 3 and Table S2).

Of the 478 SNPs, 288 were used to genotype an additional collection of 144 pear accessions in the Fluidigm assay (Figure S1); 137 accessions (92.0%) were genotyped with >90% of call rates, and 7 accessions (4.7%) were genotyped with >75% of call rates. In these accessions, 232 of the 288 SNPs (80.6%) showed reliable polymorphisms, and 9 SNPs (3.1%) were monomorphic (Table 4 and Table S3). However, the genotypes of 47 SNPs were undetermined due to ambiguous clustering patterns (31 SNPs) or no call (16 SNPs).

### 2.2. Genetic Differentiation and Diversity in the Collection of Cultivated Pears

**Figure 1:**
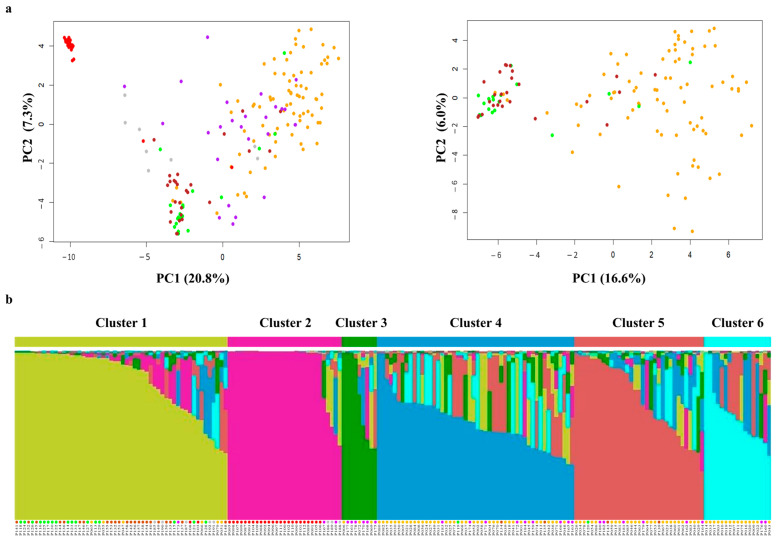
Assessment of genetic differentiation and diversity in the 192 cultivated pear accessions using 232 SNP markers. Each pear accession is indicated with colored dots: *Pyrus* pyrifolia (orange), *P. ussuriensis* (brown), *P. bretschneideri* (green), *P. communis* (red), interspecific hybrids (purple), and others (gray). (**a**) Principal component analysis of all accessions (left) and a subset for three Asian species (right). (**b**) Inferred population structure based on the model-based clustering (STRUCTURE v2.3.4) in the 192 accessions. Each accession is presented by a single vertical line, which is partitioned into six colored segments in estimated membership proportion.

The genotypic data of 232 SNPs were used to investigate genetic variations in the 192 pear accessions including four major cultivated species (*P. pyrifolia*, *P. ussuriensis*, *P. bretschneideri*, and *P. communis*) and interspecific hybrids. In a principal component analysis (PCA), PC1 and PC2 explained 20.8% and 7.3% of the total variance. These PCs separated the European pear species (*P. communis*) from the three Asian species (*P. pyrifolia*, *P. ussuriensis*, and *P. bretschneideri*) and interspecific hybrids (Figure 1a). A subsequent PCA, which was performed with the three Asian species, supported the genetic differentiation of *P. pyrifolia* from the other two species. However, the *P. ussuriensis*, and *P. bretschneideri* accessions showed no distinct separation (Figure 1a). In addition, the *P. pyrifolia* and interspecific hybrid accessions showed broad genetic diversity relative to the other species. The model-based clustering analysis showed that the 192 accessions were divided into six clusters (Figure 1b and Table S4). The first cluster (cluster 1) represented two Asian pear species, *P. ussuriensis* (23 accessions) and *P. bretschneideri* (17 accessions), with small numbers of other species (6 *P. pyrifolia*, 2 *P. communis*, 1 interspecific hybrid, and 5 others). In cluster 2, we found 24 of 26 *P. communis*, 2 interspecific hybrids, and 3 others, while only 9 accessions (6 interspecific hybrids and 3 *P. pyrifolia*) were grouped in cluster 3 (Figure 1b). The other *P. pyrifolia* and interspecific hybrid accessions were distributed into cluster 4 (38 *P. pyrifolia* and 9 interspecific hybrids), cluster 5 (24 *P. pyrifolia* and 6 interspecific hybrids), and cluster 6 (15 *P. pyrifolia* and 2 interspecific hybrids). Two *P. ussuriensis* accessions and one *P. bretschneideri* accession were found in both clusters 4 and 5.

Pairwise *F*_st_ and Nei’s genetic distance (D) were calculated to measure the magnitude and significance of genetic differentiation between the five predefined populations, representing *P. pyrifolia*, *P. ussuriensis*, *P. bretschneideri*, *P. communis*, and interspecific hybrids. All three Asian pear populations were significantly differentiated from the European pear population by pairwise *F*_st_ at *p* < 0.001 (Table 5). The highest level of genetic differentiation was found between the *P. bretschneideri* and *P. communis* populations (*F*_st_ = 0.477 and D = 0.210). The Asian pear populations showed relatively lower *F*_st_ (0.021–0.117) and D (0.016–0.103) from each other. The interspecific hybrids showed significant differentiations at *p* < 0.001 relative to *P*. *communis* (*F*_st_ = 0.371 and D = 0.193), *P. ussuriensis* (*F*_st_ = 0.059 and D = 0.040), and *P. bretschneideri* (*F*_st_ = 0.078 and D = 0.058). However, the pairwise estimates of *F_st_* and D indicated that the interspecific hybrids were more similar to *P. pyrifolia* (*F*_st_ = 0.029 and D = 0.028) relative to other species (Table 5). Allelic richness (A), expected heterozygosity (He), and PIC were also used to evaluate genetic diversity in these predefined populations. The *P. pyrifolia* and interspecific hybrid populations revealed higher estimates of A (2.23 and 2.20), He (0.45 and 0.44), and PIC (0.33 and 0.32) relative to the *P. ussuriensis* (A = 1.97, He = 0.31, and PIC = 0.26) and *P. bretschneideri* (A = 1.93, He = 0.29, and PIC = 0.25) populations (Table 6). In contrast, we found the lowest estimates (A = 1.46, He = 0.08, and PIC = 0.07) in the *P. communis* population.

### 2.3. Development of SNP Sets for Variety Identification

**Figure 2:**
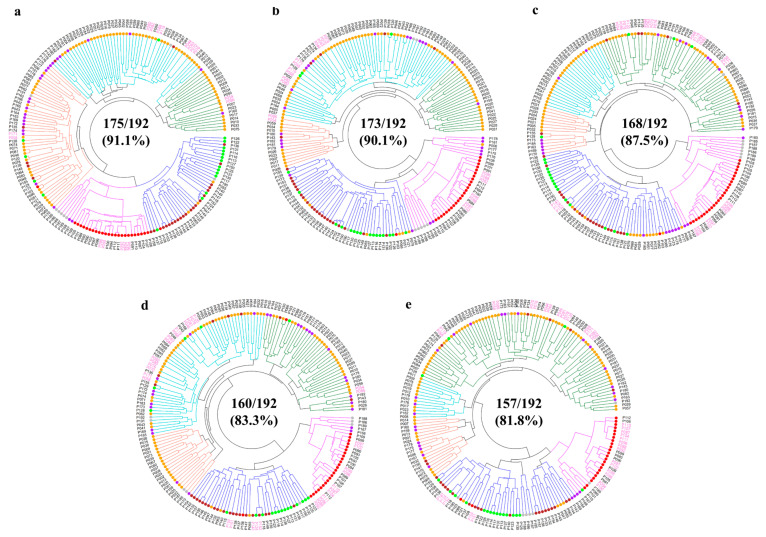
The UPGMA dendrogram of 192 cultivated pear accessions based on the Euclidean genetic distances calculated using the 232 SNP markers and four subsets: (**a**) 232, (**b**) 192, (**c**) 96, (**d**) 48, and (**e**) 24 SNPs. Each pear accession is indicated with colored dots: *Pyrus pyrifolia* (orange), *P. ussuriensis* (brown), *P. bretschneideri* (green), *P. communis* (red), interspecific hybrids (purple), and others (gray). The five major clades are shown with different colors, and undistinguished accessions are highlighted in pink.

**Figure 3:**
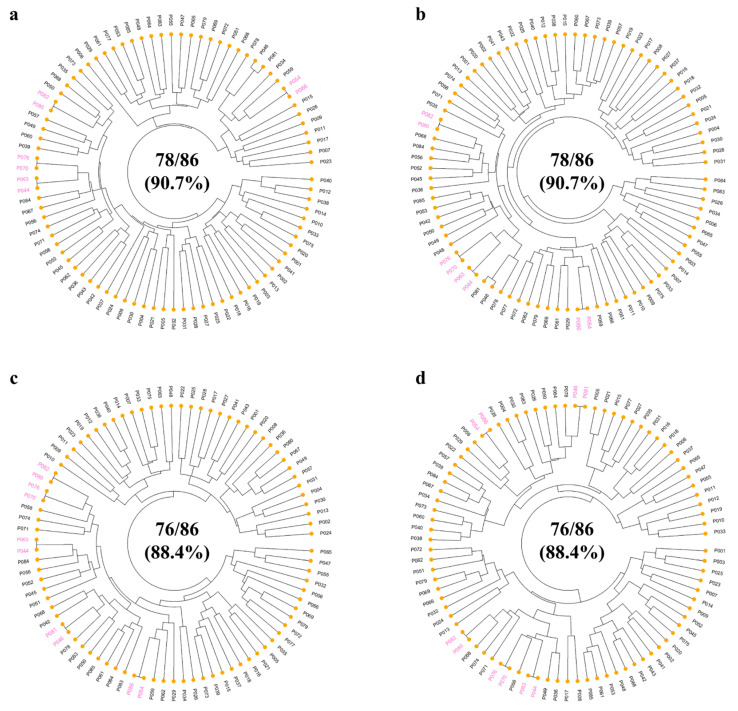
The UPGMA dendrogram of 86 *Pyrus pyrifolia* accessions based on the Euclidean genetic distances calculated using the four subsets of SNP markers: (**a**) 192, (**b**) 96, (**c**) 48, and (**d**) 24. The undistinguished accessions are highlighted in pink.

The UPGMA dendrogram based on the Euclidean genetic distance indicated that the 232 SNP markers were effective in detecting genetic variations for distinguishing 175 of 192 pear accessions (91.1%) (Figure 2a). The 17 accessions, which were undistinguished, consisted of 10 *P. pyrifolia*, 5 *P. communis*, 1 *P. bretschneideri*, and 1 interspecific hybrid. Of these, we found insufficient genetic variations to distinguish eight *P. pyrifolia* accessions: ‘Taihaku’ vs. ‘Jangseongcheongbae’, ‘Nansui’ vs. ‘Kimizukawase’, ‘Sagami’ vs. ‘Gongryong’, and ‘Goldnijisseiki’ vs. ‘Osanijisseiki’. Two *P. pyrifolia* accessions (‘Tosa’ and ‘Shinseiki’) showed no separation to ‘Jinshiji’ (*P. bretschneideri*) and ‘Shichiho’ (interspecific hybrid), respectively. For *P. communis*, three accessions (‘Harland’, Bartlett-Rosired’, and ‘Bartlett-Max Red’) were undifferentiated from each other as two accessions (Bosc-OP-5 and Bosc). From the 232 markers, four subsets of 192, 96, 48, and 24 SNPs were generated for variety identification based on their PIC values (>0.25) and distribution across 17 chromosomes. The 192 and 96 SNP sets identified 173 (90.1%) and 168 accessions (87.5%), while the 48 and 24 SNP sets differentiated 160 (83.3%) and 157 (81.8%) accessions, respectively (Figure 2b–e). Within the 86 *P. pyrifolia* accessions, these SNP sets also showed higher identification rates, which were 90.7% (192 and 96 SNPs) and 88.4% (48 and 24 SNPs), relative to those in all 192 accessions (Figure 3).

## 3. Discussion

As a member of the Rosaceae family, pear (*Pyrus* spp.) is a major fruit crop, and five commonly cultivated species are *P. communis*, *P. pyrifolia*, *P. ussuriensis*, *P. bretschneideri*, and *P. sinkiangensis* [2]. Of these, *P. communis* is known as a European pear species, while the other four species are mostly cultivated in Asia, especially China, Korea, and Japan [3]. In the present study, genotyping by sequencing was conducted for a large-scale SNP discovery in the 48 pear accessions representing a major cultivated species (*P. pyrifolia*) and interspecific hybrids. A total of 256,538 confident SNPs were found across all 17 chromosomes, and the number of SNPs per chromosome ranged from 9541 (chromosome 4) to 23,827 (chromosome 15). The GBS method was previously used to identify genome-wide SNPs in the cultivated pear species [9,11]. In 214 pear accessions including 112 *P. communis*, about 15,000 high-quality SNPs were detected from GBS to investigate genetic diversity and selection footprints [9]. Similarly, GBS of 231 pear accessions generated 10,186 SNPs for the assessment of genetic relationships and population structure [11]. Our results demonstrated that GBS is a cost-effective option to identify high-density SNPs across the genome in cultivated pear species.

A core set from the genome-wide SNPs was used to genotype an additional 144 pear accessions (53 *P. pyrifolia*, 27 *P. ussuriensis*, 19 *P. bretschneideri*, 26 *P. communis*, 14 interspecific hybrids, and 5 others) in the Fluidigm assay. The resulting genotypic data of 232 SNPs from both GBS and the Fluidigm assay revealed genetic variations in the 192 pear accessions. We found significant genetic differentiation between European and Asian pears, suggesting low levels of gene flow and introgression between these pear groups. In fact, wild European and Asian pears diverged from an ancient *Pyrus* and have undergone independent domestication processes, leading to distinct selective sweeps. In addition, *P. communis* showed narrow genetic diversity relative to the Asian species. This finding is consistent with the results of previous studies [9,11,22]. Among the Asian pears, the *P. ussuriensis* and *P. bretschneideri* accessions were clustered together, while the *P. pyrifolia* accessions were mostly separated from these species. This clustering between species was found in previous studies [11,23]. However, Wu et al. [22] suggested that the wild *P. pyrifolia* is a common ancestor for the cultivated *P. pyrifolia* and *P. bretschneideri*. Liu et al. [24] showed that *P. ussuriensis* was genetically differentiated from both *P. bretschneideri* and *P. pyrifolia*. This discrepancy could be due to sampling bias in the collections of pear accessions. We also found further separation of the *P. pyrifolia* accessions into at least three clusters, indicating high levels of genetic diversity. This result was supported by allelic richness [18,19], expected heterozygosity [20], and polymorphic information content [21]. In addition, the interspecific hybrids in this study were mostly clustered with *P. pyrifolia*, which was used as one parent, and also showed comparable genetic diversity to this parental species.

Molecular markers have been considered as an efficient tool to facilitate DUS testing in the plant variety protection (PVP) system [4,25,26]. A number of SSR markers have been developed to identify commercial cultivars in vegetable crops [25,27,28,29]. With high-throughput genotyping platforms, SNPs have been the choice of marker for variety identification. In this study, the 232 markers from 256,538 SNPs were able to separate 175 (91.1%) of 192 pear accessions. The undistinguished accessions represented 10 *P. pyrifolia*, 5 *P. communis*, 1 *P. bretschneideri*, and 1 interspecific hybrid. Of these, the *P. pyrifolia* accession ‘Nansui’ has the genetic background of ‘Kimizukawase’ that is a grandparent for this accession. The other two *P. pyrifolia* accessions ‘Goldnijisseiki’ and ‘Osanijisseiki’ are known to be somatic mutations of ‘nijisseiki’. For the *P. communis* accessions, ‘Bartlett-Max Red’ and ‘Bartlett-Rosired’ are bud mutations of ‘Bartlett’. For the remaining accessions, there is a lack of information to explain their genetic relationships, and a possible explanation is the use of a few elite parents and natural mutations leading to the development of different cultivars within breeding programs. Thus, variety identification in the pears with high levels of genetic similarity is often challenging. Since we identified 256,538 confident SNPs from GBS, it is possible to find additional markers that can detect low genetic variations in the unidentified pear accessions.

The 232 SNP markers are a powerful tool for variety identification in cultivated pears, but their subsets can also be useful to provide additional options for genotyping in different platforms. With four subsets of 192, 96, 48, and 24 SNPs, we evaluated their performances for the variety identification. The 192 SNP subset identified 173 (90.1%) of 192 pear accessions, which was only two less than the 175 accessions identified with the 232 SNP markers. Therefore, the 192 SNP markers can be considered to be a core set for variety identification in pears. In addition, the 24 SNP markers separated the European species from three Asian species and distinguished 81.8% of 192 pear accessions, suggesting that this subset is the most cost-effective option for pre-screening tests with large sample sizes. Therefore, these subsets of SNP markers are a useful resource for developing a DNA-based pipeline to protect breeders’ intellectual rights in cultivated pears.

In conclusion, the GBS-based SNP discovery in the present study identified 256,538 high-quality SNPs that can expand the genomic resources for genetics and breeding of cultivated pears. Our results also revealed that the 232 SNP markers and their subsets were effective in investigating genetic variations between and within the five populations representing four species (*P. pyrifolia*, *P. ussuriensis*, *P. bretschneideri*, and *P. communis*) and interspecific hybrids. Furthermore, these marker sets will provide a rapid and accurate approach for variety identification and facilitate the development of a DNA barcoding system for DUS testing in the PVP system. Potential applications of this molecular tool include seed purity tests and background selection in pear breeding programs.

## 4. Materials and Methods

### 4.1. Plant Materials and DNA Isolation

A total of 192 pear accessions were collected from the National Agrobiodiversity Center in Rural Development Administration, the Republic of Korea (ROK). This collection consisted of 86 *P. pyrifolia* (Burm.) Nak., 27 *P. ussuriensis* Maxim., 19 *P. bretschneideri* Rehder, 26 *P. communis* L., 3 *P. pashia* Buch.-Ham. ex D. Don, 2 *P. betulifolia* Bunge, 1 *P. sinkiangensis* T.T.Yu, 26 interspecific hybrids, and 2 unknown accessions (Table S1). These originated from at least nine countries including ROK, Japan, China, USA, and Italy. Of these, 48 pear accessions were used for genotyping by sequencing (GBS) as an SNP discovery panel, consisting of 33 *P. pyrifolia*, 12 interspecific hybrids (8 *P. pyrifolia* × *P. ussuriensis*, 3 *P. pyrifolia* × *P. communis*, and 1 *P. pyrifolia* × *P. bretschneideri*), 2 *P. pashia*, and 1 *P. betulifolia* (Table S1). For the Fluidigm assay, we used an additional 144 accessions (53 *P. pyrifolia*, 27 *P. ussuriensis*, 19 *P. bretschneideri*, 26 *P. communis*, 14 interspecific hybrids, 1 *P. pashia*, 1 *P. betulifolia*, 1 *P. sinkiangensis*, and 2 unknown).

Genomic DNA for the pear accessions was isolated from fresh, young leaves using a modified cetyl trimethyl ammonium bromide (CTAB) method [30]. The isolated DNA pellets were resuspended with the T1/10E buffer (10 mM Tris-HCl pH 8.0, 1 mM EDTA), and the final concentration of DNA was adjusted to 50 ng/μL ng for GBS and the Fluidigm assay using a NanoDrop 1000 spectrophotometer (ThermoFisher Scientific, Wilmington, DE 19810, USA).

### 4.2. Genome-Wide SNP Discovery

The GBS of 48 pear accessions was conducted according to the protocol described by Elshire et al. [31]. To construct the GBS library, 200 ng of genomic DNA for each accession was digested using a methylation-sensitive restriction enzyme, ApeKI (NEB, Ipswich, MA, USA). The DNA fragments were ligated to accession-specific barcode adapters for multiplexing and then amplified by PCR. The resulting GBS libraries were sequenced with the paired-end method in the HiSeq X platform (Illumina Inc., San Diego, CA, USA). For SNP calling, the filtered, high-quality sequencing reads were mapped to the reference genome assembly v1.0 of *P. pyrifolia* [16] using the Burrows–Wheeler Alignment (BWA) method [32]. The resulting bi-allelic SNPs with 3 x of minimum depth were filtered based on >5% of minor allele frequency and <30% of missing data. Imputation of the remaining missing SNP data was performed using BEAGLE v5.4 with default parameter settings [33] for further analysis.

### 4.3. Fluidigm Assay

A subset of SNPs was generated from the GBS-derived SNPs to genotype 144 pear accessions in the Fluidigm Juno^TM^ system (Fluidigm, San Francisco, CA, USA). For this subset, the genome-wide SNPs were filtered using polymorphism information content (PIC) and the physical map position on 17 chromosomes. The PIC value of each SNP was calculated using the following equation:

$$\mathrm{PIC}=1-\sum_{i=1}^{n}p_{i}^{2}-\sum_{i=1}^{n-1}\sum_{j=i+1}^{n}2p_{i}^{2}p_{j}^{2}$$

where n is the number of alleles and pi is the frequency of the ith allele [21].

Three types of primers were designed for the Fluidigm genotyping using the 300 bp flanking sequence of each SNP in the D3 Assay Design software (Fluidigm, San Francisco, CA, USA). Both specific target amplification and locus-specific primers were used for pre-amplification, and then two allele-specific primers were used for PCR amplification in the Juno 96.96 Genotyping IFC (Integrated Fluidic Circuit). Fluidigm SNP genotyping analysis software v4.5.1 was used to analyze the resulting end-point fluorescence images for SNP calling.

### 4.4. Data Analysis

The genotypic data from GBS and Fluidigm genotyping were used to investigate genetic variation in the collection of 192 pear accessions (Table S1). To assess genetic differentiation and diversity in these accessions, a principal component analysis (PCA) was conducted using the R package ‘pcaMethods’ [34]. In addition, population structure was inferred using the model-based clustering method as implemented in STRUCTURE v2.3.4 [35]. A series of Ks (number of clusters) from 1 to 10 was tested in 10 independent simulations per K with a burn-in of 200,000 iterations and a run length of 500,000 iterations. The best K was determined using the delta K method in Structure Harvester v0.6.94 [36,37], and the membership coefficients of 192 accessions were visualized using Structure Plot v2.0 [38]. For hierarchical clustering analysis, the Euclidean genetic distance was estimated between pear accessions using the R package ‘poppr’ [39], and then the unweighted pair group method with arithmetic mean (UPGMA) dendrogram was generated using the R package ‘dendextent’ [40]. Furthermore, pairwise *F*_st_ and Nei’s genetic distance were calculated between predefined populations based on species in the 184 pear accessions Microsatellite Analyzer (MSA) v4.05 [41]. For this analysis, we excluded the eight accessions of three species (1 *P. sinkiangensis*, 2 *P. betulifolia*, and 3 *P. pashia*) and 2 unknown species due to small sample sizes. The *p*-value for the pairwise estimate of *F*_st_ was obtained from 10,000 permutations of genotypes and a Bonferroni correction was applied [17]. Allelic richness [18,19] and expected heterozygosity [20] in each population were also estimated with the MSA.

## Figures and Tables

**Table 1:** Summary of genotyping by sequencing (GBS) in the 48 cultivated pear accessions.

Illumina Pair-End Sequencing	
No. of raw reads	774,122,148
Average length of raw reads (bp)	151
Total length of raw reads (Gb)	116.9
No. of demultiplexed reads	696,225,442
Average length of trimmed reads (bp)	123.50
Total length of trimmed reads (Gb)	80.50
No. of mapped reads	647,930,904
Percentage of mapped reads (%)	97.9
Average length of mapped regions (bp)	312.90
Total length of mapped regions (Gb)	2.8
No. of total SNPs	2,493,326
No. of filtered SNPs ^a^	256,538

^a^ Bi-allelic SNPs across the 17 pear chromosomes passed three criteria: >5% of the minor allele frequency, <15% of the missing data rate, and 3× of the minimum depth.

**Table 2:** Distribution of 256,538 confident SNPs on 17 pear chromosomes.

Chromosome	No. of SNP	Coverage (Mb) ^a^	Marker Interval (Mb)	
			Maximum	Average
1	14,738 (19) ^b^	23.12 (18.98)	0.20 (5.28)	0.002 (1.05)
2	16,891 (27)	31.75 (31.01)	0.39 (5.04)	0.002 (1.19)
3	14,978 (30)	31.60 (29.61)	0.17 (4.86)	0.002 (1.02)
4	9541 (22)	19.46 (18.94)	0.20 (4.26)	0.002 (0.90)
5	15,246 (22)	35.23 (33.05)	0.31 (7.35)	0.002 (1.57)
6	14,505 (29)	22.02 (21.21)	0.30 (2.49)	0.002 (0.76)
7	13,144 (33)	36.07 (34.83)	0.56 (5.03)	0.003 (1.09)
8	13,425 (37)	26.49 (26.21)	0.21 (3.10)	0.002 (0.73)
9	13,680 (29)	24.99 (24.20)	0.19 (4.03)	0.002 (0.86)
10	12,462 (19)	20.99 (14.42)	0.23 (2.85)	0.002 (0.80)
11	17,273 (35)	35.07 (33.38)	0.34 (5.61)	0.002 (0.98)
12	15,373 (25)	24.61 (24.03)	0.46 (5.22)	0.002 (1.00)
13	15,727 (22)	29.54 (28.36)	0.14 (5.45)	0.002 (1.35)
14	14,834 (24)	22.55 (22.11)	0.96 (2.69)	0.002 (0.96)
15	23,827 (47)	39.62 (39.33)	0.25 (2.94)	0.002 (0.85)
16	13,035 (28)	25.45 (25.08)	0.22 (2.95)	0.002 (0.93)
17	17,859 (30)	31.98 (29.89)	0.34 (4.78)	0.002 (1.03)
Total	256,538 (478)	480.54 (454.64)	0.96 (7.35)	0.002 (0.99)

^a^ Physical positions of SNPs were determined based on the *Pyrus pyrifolia* genome assembly v1.0 [16]. ^b^ The number in parentheses indicates the SNPs obtained by filtering 256,538 SNP markers with three criteria (>0.25 of the polymorphic information content value, <35% of the heterozygosity rate, and a physical position of the *P. pyrifolia* genome).

**Table 3:** The subsets of genome-wide SNPs for validation and core marker selection.

Class ^a^		No. of SNP ^b^
Coding sequence variant		326 (243)
Non-coding sequence variant	Intron variant	96 (35)
	Intergenic variant	56 (10)
Total		478 (288)

^a^ This is based on the annotation of the *Pyrus pyrifolia* genome assembly v1.0 [16]. ^b^ Number in the parentheses indicates SNPs used for the Fluidigm assay in an additional collection of 144 pear accessions.

**Table 4:** Polymorphism of 288 SNPs in an additional 144 pear accessions used for the Fluidigm assay.

Class	No. of Markers	Percentage (%)
Polymorphic	232	80.6
Monomorphic	9	3.1
Undetermined ^a^	31	10.8
No call	16	5.6
Total	288	100

^a^ Polymorphism detected but ambiguous genotype calls or high percentages of missing data (>30%).

**Table 5:** Pairwise estimates of *F*_st_ and Nei’s genetic distance between the pear populations based on 232 SNP markers.

Predefined Population ^a^	Sample Size	*P. pyrifolia*	*P. ussuriensis*	*P. bretschneideri*	*P. communis*	Interspecific Hybrid
*Pyrus pyrifolia*	86		0.110 ^b,^**	0.117 **	0.352 **	0.029 **
*P. ussuriensis*	27	0.092		0.021 *	0.410 **	0.059 **
*P. bretschneideri*	19	0.103	0.016		0.477 **	0.078 **
*P. communis*	26	0.297	0.186	0.210		0.371 **
Interspecific hybrid	26	0.028	0.040	0.058	0.193	

^a^ One *P. sinkiangensis*, two *P. betulifolia*, three *P. pashia*, and two unknown accessions were excluded from this analysis. ^b^ Pairwise estimates of *F*_st_ (upper right diagonal) between populations and Nei’s standard genetic distance corrected for sample size (lower left diagonal); *p*-value was calculated by 10,000 permutations with a Bonferroni correction [17]. * *p* < 0.005 and ** *p* < 0.001.

**Table 6:** Descriptive statistics for genetic diversity within the pear populations based on 232 SNP markers.

Predefined Population ^a^	Sample Size	A ^b^	He ^c^	PIC ^d^
*Pyrus pyrifolia*	86	2.23	0.45	0.33
*P. ussuriensis*	27	1.97	0.31	0.26
*P. bretschneideri*	19	1.93	0.29	0.25
*P. communis*	26	1.46	0.08	0.07
Interspecific hybrid	26	2.20	0.44	0.32
Total	184	2.22	0.31	0.32

^a^ One *P. sinkiangensis*, two *P. betulifolia*, three *P. pashia*, and two unknown accessions were excluded from this analysis. ^b^ Allelic richness [18,19]. ^c^ Expected heterozygosity [20]. ^d^ Polymorphism information content [21].

## Data Availability

All data are provided in the article and Supplementary Materials.

## Supplementary Materials

The following supporting information can be downloaded at: https://www.mdpi.com/article/10.3390/plants13182600/s1, Table S1: The 192 cultivated pear accessions used in this study; Table S2: The predicted functions of genes associated with 326 SNPs based the *Pyrus pyrifolia* v1.0 genome assembly, Table S3: The genotypes of 232 SNP markers in the 192 pear accessions, Table S4: The membership coefficients of 192 pear accessions and their clusters at the best K (K = 6) in the STRUCTURE analysis using 232 SNP markers; Figure S1: Distribution of SNPs in two subsets from 256,538 genome-wide confident SNPs on 17 pear chromosomes. (A) Physical positions of 478 SNPs and (B) 288 SNPs based on the *Pyrus pyrifolia* genome assembly v1.0 [16]. The horizontal lines with different color codes indicate SNPs from coding (red), intron (green), and intergenic sequences (blue). The number of SNPs per chromosome is present below each bar.
