# Supplementary material for: Development of Cost-Effective SNP Markers for Genetic Variation Analysis and Variety Identification in Cultivated Pears (Pyrus spp.)
Source: Plants (Basel). 2024 Sep 18;13(18):2600. doi: 10.3390/plants13182600 (PMC11435430; doi:10.3390/plants13182600)
Supplement: Supplementary file 1 [file plants-13-02600-s001.zip › plants-3166604-supplementary/Figure S1.pdf]

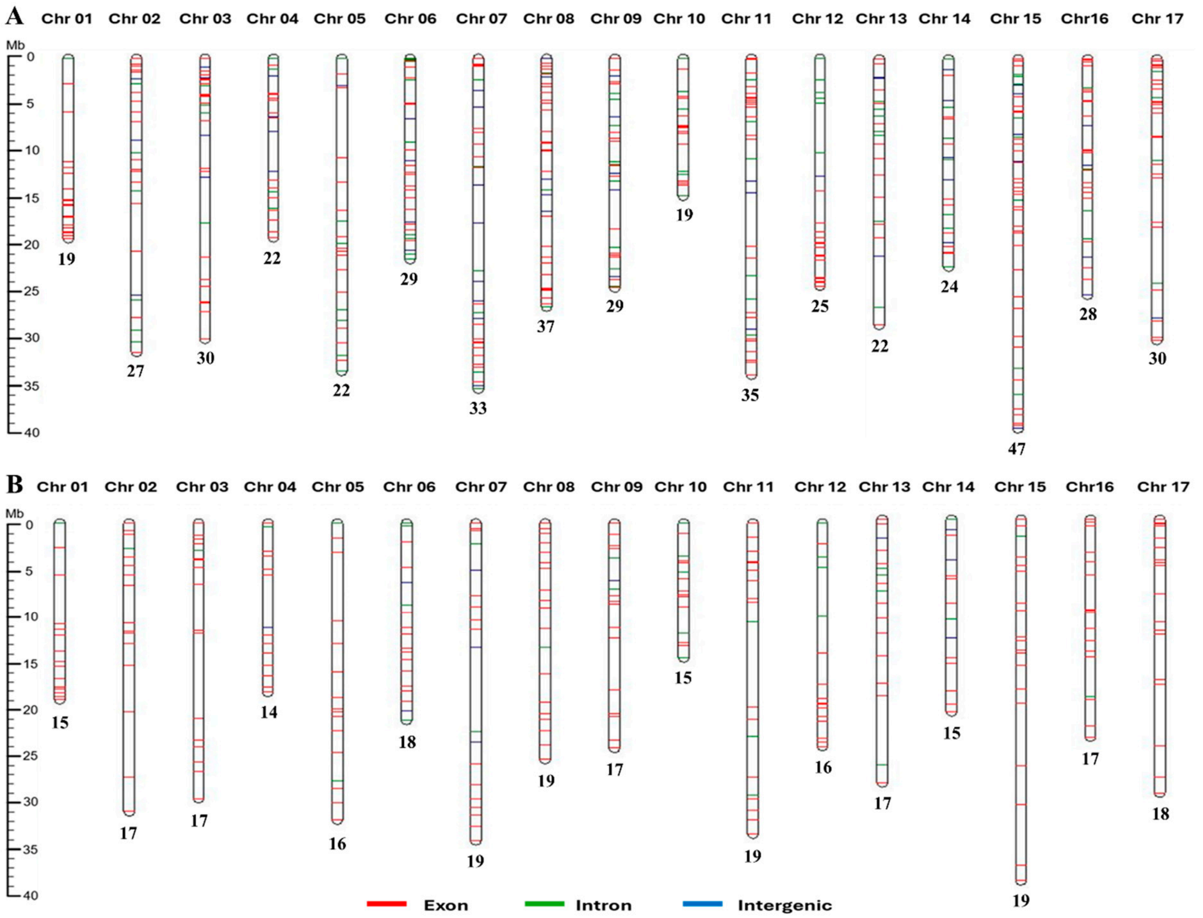

Figure S1: Distribution of SNPs in two subsets from 256,538 genome-wide confident SNPs on 17 pear c hromosomes. (A) Physical positions of 478 SNPs and (B) 288 SNPs based on the *Pyrus pyrifolia* genome assembly v1.0 [16]. The horizontal lines with different color codes indicate SNPs from coding (red), int ron (green), and intergenic sequences (blue). The number of SNPs per chromosome is present below e ach bar.
